# GSDMD induces hepatocyte pyroptosis to trigger alcoholic hepatitis through modulating mitochondrial dysfunction

**DOI:** 10.1186/s13008-024-00114-0

**Published:** 2024-03-26

**Authors:** Yandi Xie, Zilong Wang, Guangjun Song, Hui Ma, Bo Feng

**Affiliations:** Peking University People’s Hospital, Peking University Hepatology Institute, Beijing Key Laboratory of Hepatitis C and Immunotherapy for Liver Diseases, Beijing International Cooperation Base for Science and Technology on NAFLD Diagnosis, No.11, Xizhimen South Street, Beijing, 100044 China

**Keywords:** GSDMD, Alcoholic hepatitis, Mitochondrial dysfunction, Drp1

## Abstract

**Background:**

Mechanisms and consequences of Gasdermin D (GSDMD) activation in alcoholic hepatitis (AH) are unclear. In the present study, we investigated whether GSDMD induces hepatocyte pyroptosis by regulating mitochondrial dysfunction in AH.

**Results:**

Liver damage in AH mice was assessed by HE staining, serum levels of AST, ALT, TC, and TG. The levels of IL-1β, IL-18, LDH, inflammasome-associated proteins and hepatocyte death were assessed to determine pyroptosis. Mitochondrial dysfunction was assessed through various parameters including mitochondrial DNA (mtDNA) levels, ROS generation, mitochondrial membrane potential, ATP contents, levels of mitochondrial function-related proteins and morphological changes of mitochondria. AH induced gasdermin D (GSDMD) activation, leading to increased protein expression of N-terminal GSDMD (GSDMD-N), NLRP3, and Caspase 11 in liver tissues. Downregulation of GSDMD alleviated alcohol-induced hepatocyte pyroptosis. Alcohol also causes mitochondrial dysfunction in hepatocytes in AH, which was improved by inhibiting GSDMD. Furthermore, enhancing mitochondrial function suppressed alcohol-induced hepatocyte pyroptosis. Further, knockdown of GSDMD or dynamin-related protein 1 (Drp1) improved AH-induced liver injury, accompanied by a decrease in hepatocyte pyroptosis.

**Conclusion:**

GSDMD induces hepatocyte pyroptosis by modulating mitochondrial dysfunction during AH-induced inflammation and liver injury. These findings may pave the way to develop new therapeutic treatments for AH.

**Supplementary Information:**

The online version contains supplementary material available at 10.1186/s13008-024-00114-0.

## Background

Alcoholic liver disease (ALD) encompasses a range of liver conditions, from fatty liver (steatosis) to severe outcomes such as alcoholic hepatitis, fibrosis, and ultimately cirrhosis, all attributable to chronic alcohol consumption [1, 2]. Alcoholic hepatitis (AH) represents a critical clinical manifestation of ALD, often culminating in liver failure and associated with elevated mortality rates [3]. Among the well-documented pathogenic processes of ALD are hepatic steatosis, oxidative stress, acetaldehyde-mediated toxicity, metabolic dysregulation, and inflammation orchestrated by cytokines and chemokines. In AH, excessive ethanol directly or indirectly leads to hepatocellular damage, followed by hepatocellular death characterized by apoptosis, necrosis, or necroptosis. Alcohol-induced inflammation emerges as a pivotal feature in alcoholic steatosis, culminating in the development of alcoholic steatohepatitis (ASH). It serves as a principal driving force for fibrogenesis, ultimately leading to the progression of fibrosis, cirrhosis, and likely hepatocarcinogenesis [4]. While unfortunately, currently recommended treatments for AH, such as corticosteroids, pentoxifylline, and nutritional support, only offer limited survival benefit [5]. The cellular and molecular mechanisms underlying the pathogenesis of ALD remain incompletely understood [6]. Therefore, efforts focused on exploring the specific mechanism of AH-induced hepatocyte death are important for finding a new therapy for AH.

Growing evidence indicates that mitochondrial dysfunction plays a major role in alcohol-induced hepatocyte regeneration and liver injury [7–9]. In AH, alcohol directly or indirectly causes cell damage and the release of reactive oxygen species (ROS), subsequently triggering oxidative stress, mitochondrial damage, and dysfunction [9, 10]. Consequently, the dysfunction of mitochondria can lead to nuclear DNA damage, which contributes to cell death [8]. This, in turn, results in cell rupture and the release of damage-associated molecular patterns (DAMPs), ultimately causing inflammatory responses and liver injury. Targeting mitochondria could be a potential therapeutic approach to inhibit hepatocellular injury and death in AH.

Gasdermin D (GSDMD) has been reported as pore-forming effector proteins that cause membrane permeabilization and cell pyroptosis [11, 12]. Under systemic inflammation, both pathogen-associated molecular patterns (PAMPs) and DAMPs are recognized by immune cells and initiate the activation of Caspases. Activated Caspases cleave GSDMD, leading its activation and the generation of an N-terminal fragment (GSDMD-N). This fragment will then insert into the cell membranes, forming pores that allow the release of pro-inflammatory cytokines, such as IL-1β and IL-18. This process ultimately leads to cell rupture and a lytic pro-inflammatory form of cell death known as pyroptosis [13]. Recent published study has shown that activated GSDMD (GSDMD-N) can bind to mitochondrial membranes, causing the loss of mitochondrial membrane potential and promoting mitochondrial damage and cell pyroptosis [14]. In addition, GSDMD-mediated cell pyroptosis has been reported as a new immune regulator potentially involved in AH [15, 16]. However, the role of GSDMD activation in mediating mitochondrial dysfunction in AH-induced cell pyroptosis and liver injury is still under investigation.

In the current study, our aim was to test the hypothesis that GSDMD induces hepatocyte pyroptosis by modulating mitochondrial dysfunction during AH-induced inflammation and liver injury. To achieve this aim, we further investigated the specific mechanisms underlying mitochondrial dysfunction in hepatocytes and examined the activation of GSDMD and its downstream effects in hepatocytes exposed to alcohol, as well as in an AH mice model.

## Results

### AH induces GSDMD activation through the non-canonical inflammasome pathway

**Fig. 1:**
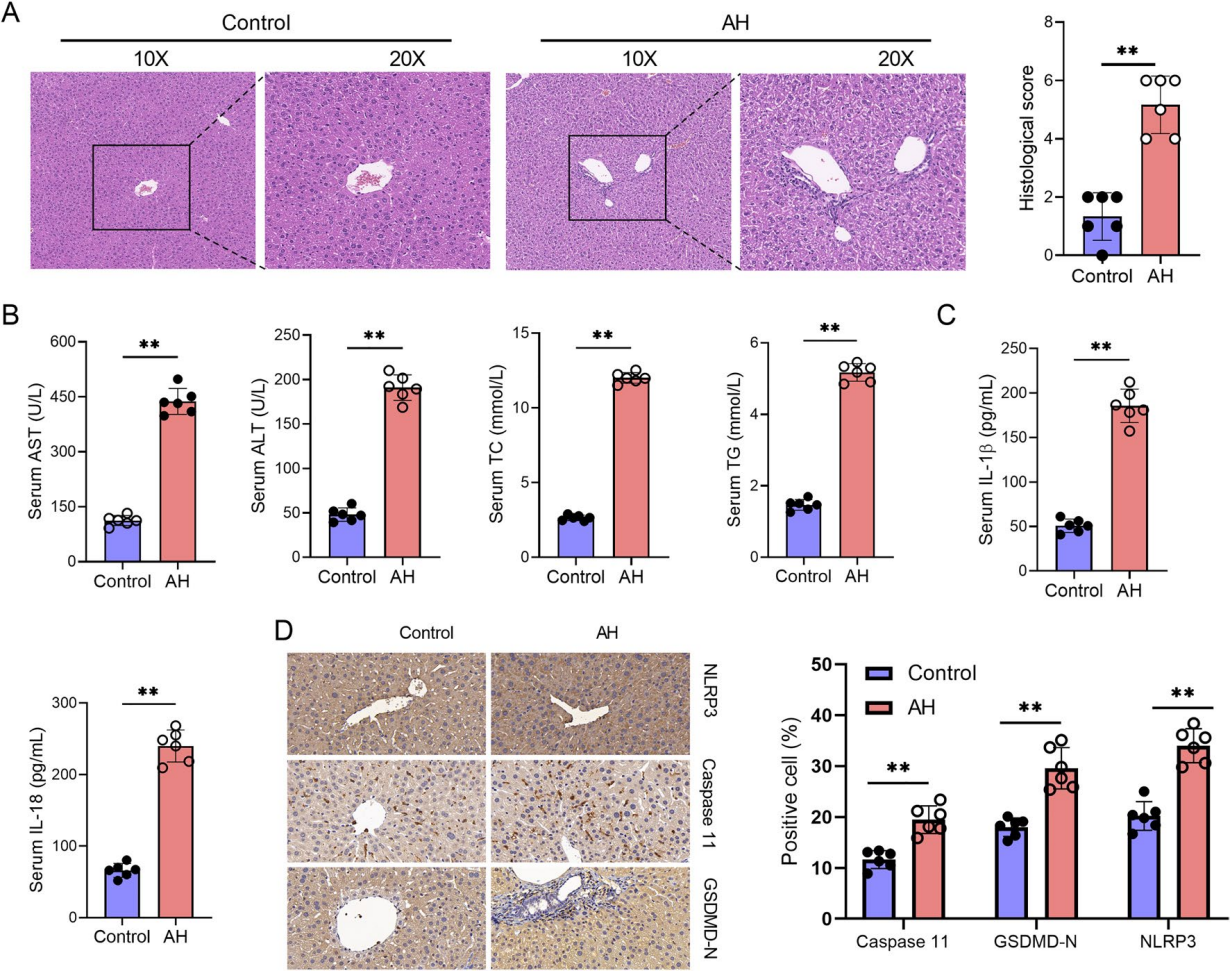
AH induces GSDMD activation through the non-canonical inflammasomes pathway. Alcoholic hepatitis (AH) mouse model was induced by administering alcohol via gavage directly into the animal's stomach in normal mice. At the endpoint of each experiment, the blood and liver tissues were collected. **A** The injury of liver tissues showed by HE staining. **B** The serum levels of AST, ALT, TC and TG in control or AH mice were measured by commercial kits. **C** The serum levels of IL-1β and IL-18 in control or AH mice were measured by ELISA kits. **D** The expression of GSDMD-N, NLRP3, and Caspase 11 in liver tissues of control or AH mice were measured by IHC. Data are presented as mean ± SD (n = 6 mice per group). Unpaired *t*-test was performed for statistical analysis. **p < 0.01

Compared with normal control mice, the HE staining of liver tissues revealed hepatocyte cytoplasmic blebbing, fatty degeneration, focal necrosis with neutrophil infiltration, and hepatic sinusoidal congestion, indicating significant liver injury in AH mice (Fig. 1A). Correspondingly, the serum levels of AST, ALT, TC and TG were significantly increased in AH mice (Fig. 1B). The results indicate that the AH mouse model was successfully induced in this study. To investigate the role of the GSDMD pathway in AH, we assessed the expression of GSDMD pathway-related proteins in the liver tissues of control and AH mice. We found that the serum levels of IL-1β and IL-18 were increased in AH mice compared to those in the control mice (Fig. 1C). Additionally, the protein expression of GSDMD-N, NLRP3, and Caspase 11 was upregulated in the liver tissues of AH mice compared to the control mice (Fig. 1D). Therefore, these results suggest that AH could induce GSDMD activation through the non-canonical inflammasome pathway.

### Downregulation of GSDMD alleviates alcohol-induced hepatocyte pyroptosis

**Fig. 2:**
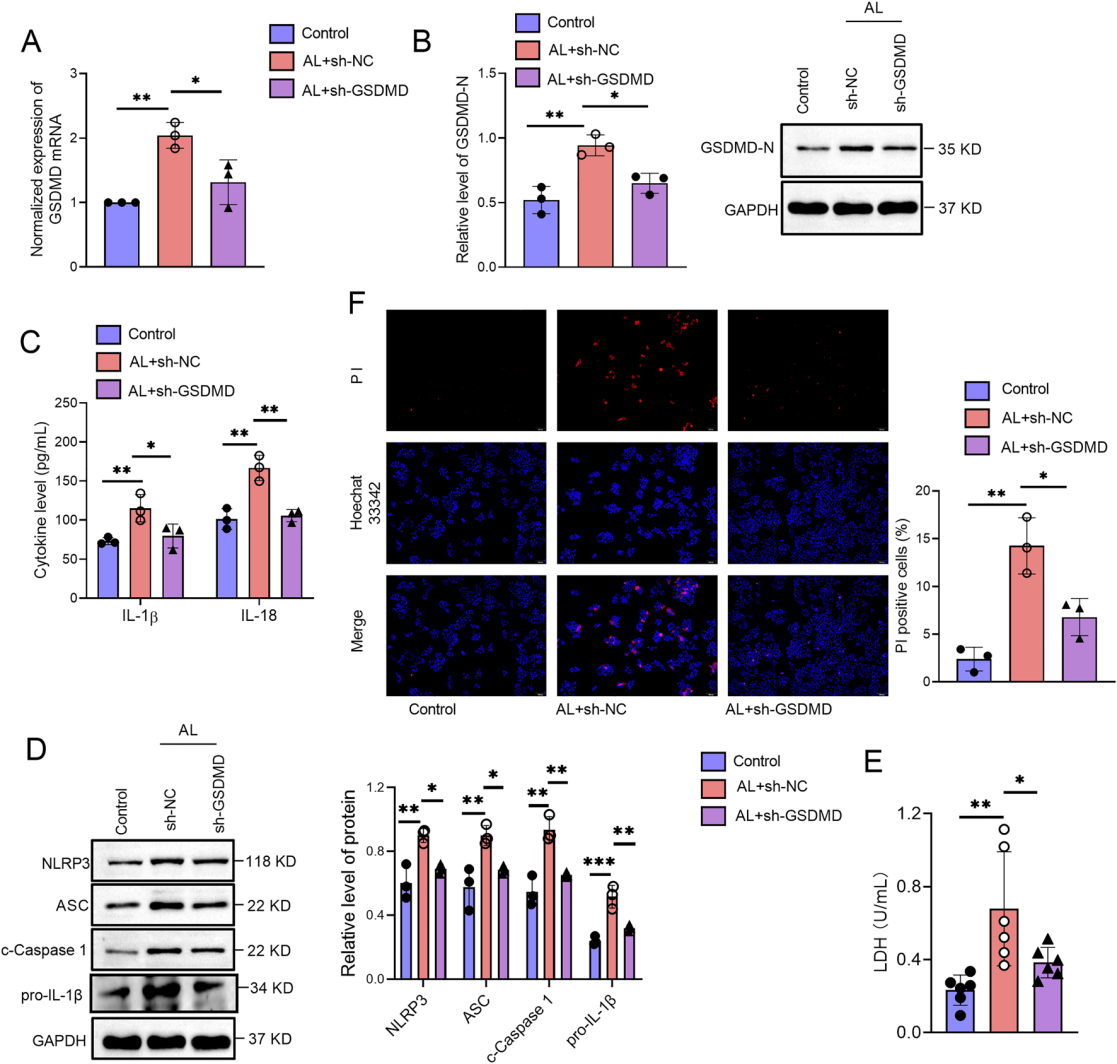
Downregulation of GSDMD alleviates alcohol-induced hepatocyte pyroptosis. GSDMD downregulation shRNA (sh-GSDMD) or negative control shRNA (sh-NC) were transfected into hepatocytes. After transfection, hepatocytes were stimulated with or without 40% alcohol for 48 h. **A** The gene expression of GSDMD in hepatocytes was determined by qPCR. **B** The protein expression of GSDMD-N in hepatocytes was determined by western blot. **C** The levels of IL-1β and IL-18 in the culture supernatants of hepatocytes were measured by ELISA kits. **D** The expressions of NLRP3, ASC, cleaved Caspase 1 and pro-IL-1β in hepatocytes were determined by western blot. **E** The LDH level in the supernatants of hepatocytes was measured. **F** Fluorescence microscopy analysis of propidium iodide (PI) internalization expressed as PI positive cells percentages (%). Data are presented as mean ± SD. *p < 0.05, **p < 0.01

To further confirm the role of GSDMD in AH, we downregulated GSDMD expression in hepatocytes. We found that alcohol upregulated the gene expression of GSDMD in hepatocytes, while sh-GSDMD significantly reversed the increase in GSDMD gene expression induced by alcohol compared with sh-NC treated hepatocytes (Fig. 2A). We also found that sh-GSDMD significantly reversed the increase in GSDMD-N levels induced by alcohol compared with sh-NC treated hepatocytes (Fig. 2B). Correspondingly, the serum levels of IL-1β and IL-18 were significantly increased by alcohol, while GSDMD knockdown partially reversed the increase induced by alcohol (Fig. 2C). We also found that sh-GSDMD suppressed the increased expression levels of NLRP3, ASC, cleavage-Caspase 1, and pro-IL-1β induced by alcohol (Fig. 2D). The LDH levels were increased in the supernatants of hepatocytes after being stimulated with alcohol. While sh-GSDMD decreased LDH levels in alcohol-treated hepatocytes compared with the sh-NC group (Fig. 2E). Further, inhibition of GSDMD could decrease PI-positive cells in alcohol-treated hepatocytes (Fig. 2F). Thus, these results indicate that downregulation of GSDMD alleviated alcohol-induced hepatocyte pyroptosis.

### Alcohol causes mitochondrial dysfunction in hepatocytes in AH

**Fig. 3:**
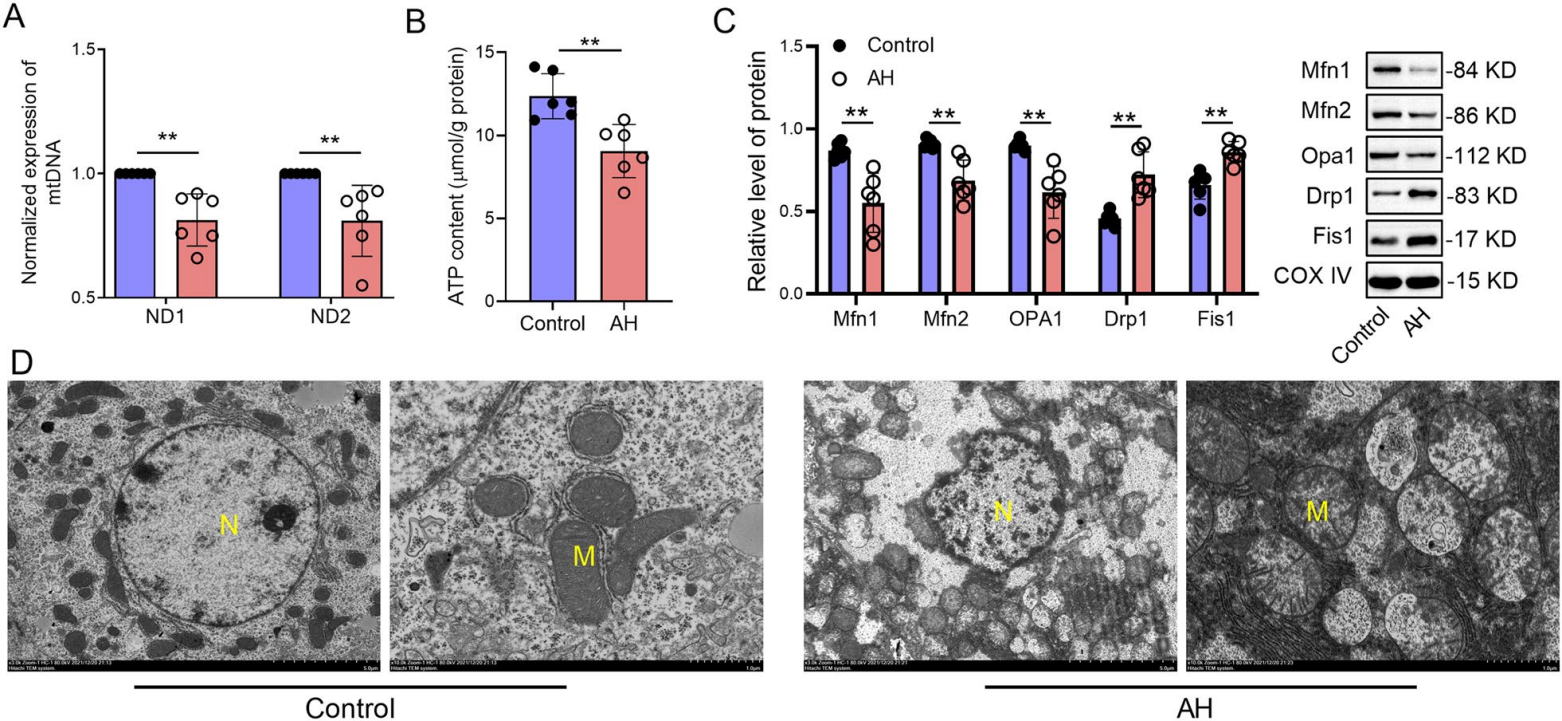
Alcohol causes mitochondrial dysfunction in hepatocytes in AH. Alcoholic hepatitis (AH) mouse model was induced by administering alcohol via gavage directly into the animal's stomach in normal mice. At the endpoint of each experiment, the blood and liver tissues were collected. **A** The gene expression of ND1 and ND2 in liver tissues of control or AH mice were measured by qPCR. **B** The level of ATP in liver tissues of control or AH mice were measured by ATP kits. **C** The expression levels of Mfn1, Mfn2, Opa1, Drp1, and Fis1 in liver tissues of control or AH mice were measured by western blot. Data are presented as mean ± SD (n = 6 mice per group). Unpaired *t*-test was performed for statistical analysis. **p < 0.01. **D** The mitochondria structure of hepatocytes in liver tissues of control or AH mice was imaged by TEM.

To investigate the role of mitochondrial dysfunction in AH, the expression of proteins related to mitochondrial function was determined by western blot analysis. We observed that the expression of the ND1 and ND2 genes in liver tissues was significantly decreased in AH mice, indicating the presence of damaged mtDNA in liver cells (Fig. 3A). We also found that the ATP level of liver tissue was reduced in AH mice compared to the control mice (Fig. 3B). Moreover, the protein expression of Mfn1, Mfn2, and Opa1 was decreased, while the protein levels of dynamin-related protein 1 (Drp1) and Fis1 were increased in the liver tissues of AH mice compared to those in the control mice (Fig. 3C). The results of transmission electron microscopy (TEM) observation showed mitochondrial swelling, vacuolization, fragmentation, or absence of mitochondrial cristae in the liver tissues of AH (Fig. 3D). Therefore, these results suggest that AH could cause mitochondrial dysfunction in hepatocytes.

### Downregulation of GSDMD improves alcohol-induced hepatocyte mitochondrial dysfunction

**Fig. 4:**
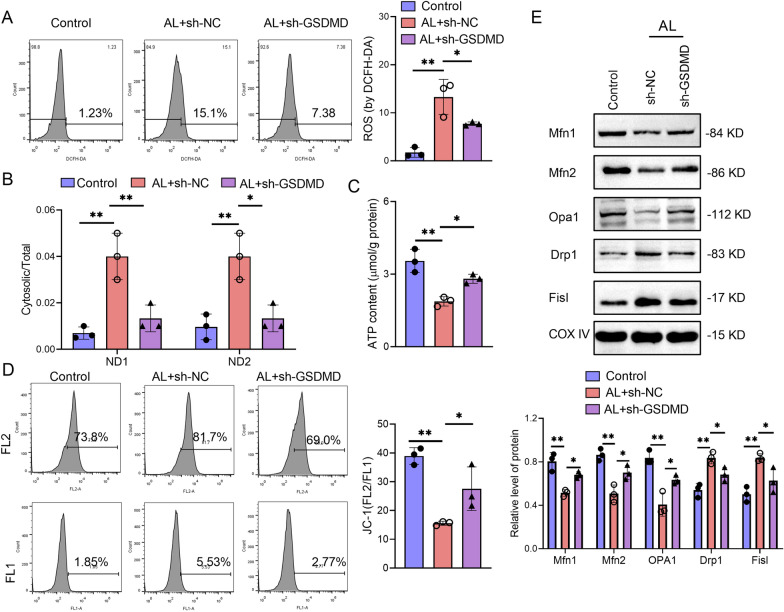
Downregulation of GSDMD improves alcohol-induced hepatocyte mitochondrial dysfunction. GSDMD downregulation shRNA (sh-GSDMD) or negative control shRNA (sh-NC) were transfected into hepatocytes. After transfection, hepatocytes were stimulated with or without 40% alcohol for 48 h. **A** Flow cytometry analysis of ROS using DCFH-DA in hepatocytes. **B** The gene expression of mtDNA (ND1 and ND2) in hepatocytes was determined by qPCR. **C** The level of ATP in hepatocytes was measured by ATP kits. **D** Mitochondrial membrane potential of cells was analyzed by Flow cytometry with by a JC-1 staining kit. **E** The protein expression levels of Mfn1, Mfn2, Opa1, Drp1 and Fis1 in hepatocytes were measured by western blot. Data are presented as mean ± SD. *p < 0.05, **p < 0.01

To further confirm the role of GSDMD in AH-induced hepatocytes’ mitochondrial dysfunction, we downregulated GSDMD expression in hepatocytes. Alcohol induced ROS production in sh-NC treated hepatocytes, while sh-GSDMD significantly inhibited the increase in ROS levels (Fig. 4A). We found that sh-GSDMD could decrease the increased cytosolic mtDNA levels (ND1 and ND2) induced by alcohol compared with sh-NC treated hepatocytes (Fig. 4B). The ATP level was significantly increased, and the mitochondrial membrane potential was decreased in alcohol-induced hepatocytes, while these effects were partly reversed by sh-GSDMD (Fig. 4C and D). Correspondingly, the expression of the proteins Mfn1, Mfn2, and Opa1 was downregulated, while the protein levels of Drp1 and Fis1 were upregulated by alcohol, while these effects were repressed by sh-GSDMD, as illustrated in Fig. 4E. Taken together, these results indicate that downregulation of GSDMD improves alcohol-induced hepatocyte mitochondrial dysfunction.

### Improving mitochondrial dysfunction suppresses alcohol-induced hepatocyte pyroptosis

**Fig. 5:**
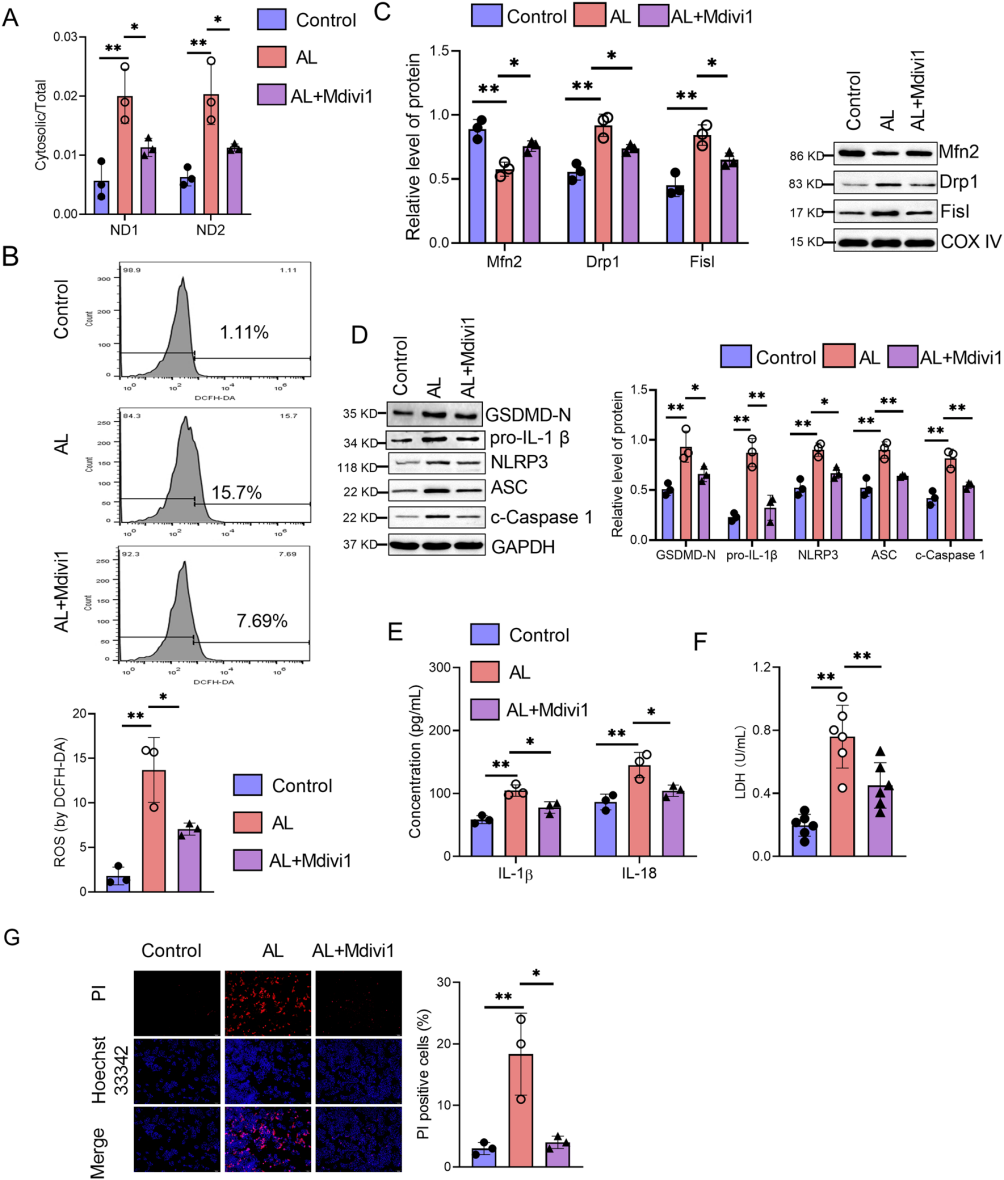
Improving mitochondrial dysfunction suppresses alcohol-induced hepatocyte pyroptosis. Hepatocytes were pretreated with 10 µM Drp1 inhibitor mdivi1 for 12 h, followed by stimulation with or without 40% alcohol. After stimulation, cells were collected for subsequent experiments. **A** The gene expression of mtDNA (ND1 and ND2) in hepatocytes was determined by qPCR. **B** Flow cytometry analysis of ROS by DCFH-DA in hepatocytes. **C** The protein expression of Mfn2, Drp1 and Fis1 in hepatocytes were measured by western blot. **D** The expression of GSDMD-N, NLRP3, ASC, cleaved Caspase 1, and pro-IL-1β in hepatocytes was determined by western blot. **E** The levels of IL-1β and IL-18 in the culture supernatants of hepatocytes were measured by ELISA kits.  **F** The LDH levels in the supernatants of hepatocytes were measured. **G** Fluorescence microscopy analysis of propidium iodide (PI) internalization expressed as PI positive cells percentages (%). Data are presented as mean ± SD. *p < 0.05, **p < 0.01

To further confirm the role of Drp1-mediated mitochondrial dysfunction in alcohol-induced hepatocyte pyroptosis in vitro, the Drp1 inhibitor mdivi1 was applied in this study. Firstly, Mdivi1 not only failed to induce hepatocyte cell injury, as evidenced by the absence of increased ROS, IL-1β and IL-18 production. (Additional file 1: Fig. S1A, B). Mdivi1 also did not affect the expression of NLRP3, ASC, and cleaved Caspase 1 in hepatocytes (Additional file 1: Fig. S1C). Mdivi1 treatment significantly decreased the cytosolic levels of mtDNA (ND1 and ND2) induced by alcohol in hepatocytes (Fig. 5A). Mdivi1 treatment also suppressed ROS production induced by alcohol in hepatocytes (Fig. 5B). The expression of the Mfn2 protein was downregulated, while the protein levels of Drp1 and Fis1 were upregulated by alcohol in hepatocytes, and these effects were partly reversed by Mdivi1 (Fig. 5C). Moreover, the upregulated levels of NLRP3, ASC, cleaved Caspase 1, GSDMD-N, and pro-IL-1β were significantly reduced by Mdivi1 treatment in alcohol-stimulated hepatocytes (Fig. 5D). Mdivi1 treatment significantly decreased the elevated levels of IL-1β and IL-18 in alcohol-induced hepatocytes (Fig. 5E). We found that the LDH levels were increased in hepatocytes after being stimulated with alcohol. While the Drp1 inhibitor Mdivi1 decreased LDH levels in alcohol-treated hepatocytes (Fig. 5F). Mdivi1 treatment also suppressed the alcohol-induced hepatocyte cell death, as evidenced by Hoechst 33342/PI staining (Fig. 5G). Together, these results suggest that improving mitochondrial dysfunction could suppress alcohol-induced hepatocyte pyroptosis.

### GSDMD mediates hepatocyte pyroptosis by regulating mitochondrial function in AH

**Fig. 6:**
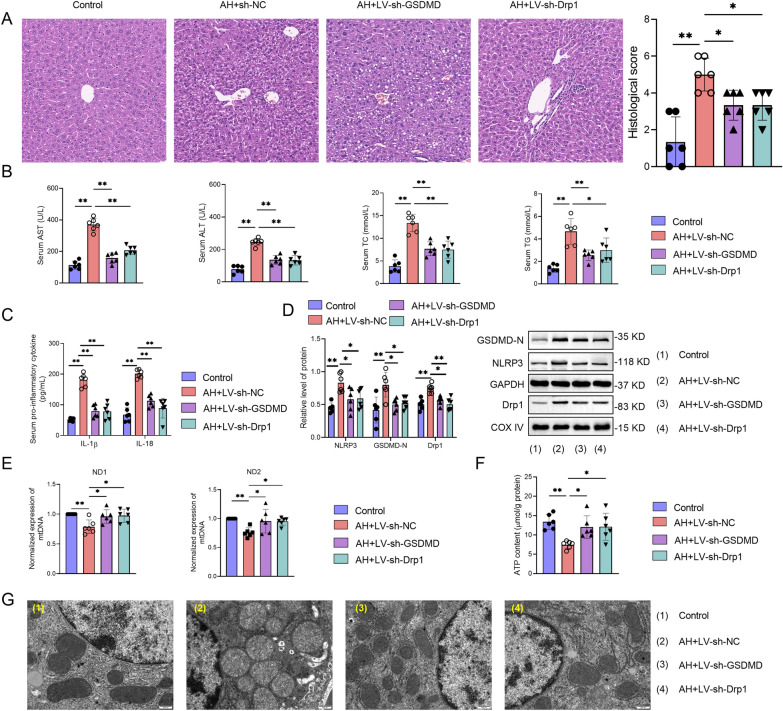
GSDMD mediates hepatocyte pyroptosis by regulating mitochondrial function in AH. GSDMD downregulation shRNA (sh-GSDMD) or Drp1 downregulation shRNA (sh-Drp1) or negative control shRNA (sh-NC) lentivirus were applied to AH mice by tail intravenous injection. At the endpoint of each experiment, the blood and liver tissues were collected. **A** The injury of liver tissues was showed by HE staining. **B** The serum levels of AST, ALT, TC and TG in control or AH mice were measured by commercial kits. **C** The serum levels of IL-1β and IL-18 in control or AH mice were measured by ELISA kits. **D** The protein expression of NLRP3, GSDMD-N, and Drp1 in liver tissues of control or AH mice was determined by western blot. **E** The gene expression of mtDNA (ND1 and ND2) in liver tissues of control or AH mice was determined by qPCR. **F** The level of ATP in liver tissues of control or AH mice were measured by ATP kits. Data are presented as mean ± SD (n = 6 mice per group). *p < 0.05, **p < 0.01. **G** The mitochondria structure of hepatocytes in liver tissues of control or AH mice were imaged by TEM.

GSDMD or Drp1 downregulation significantly improved AH-induced liver injury compared with sh-NC treated AH mice (Fig. 6A). sh-GSDMD and sh-Drp1 treatment also suppressed the serum levels of AST, ALT, TC and TG compared with sh-NC treated AH mice, respectively (Fig. 6B). Additionally, inhibition of GSDMD or Drp1 evidently decreased the elevated levels of IL-1β and IL-18 compared with sh-NC treated AH mice (Fig. 6C). The expression levels of GSDMD-N, NLRP3, and Drp1 upregulated by alcohol was partially reversed by sh-GSDMD or sh-Drp1 treatment compared with sh-NC treated AH mice (Fig. 6D). We also found that treatment with sh-GSDMD or sh-Drp1 suppressed the levels of mtDNA and ATP in liver tissues compared with sh-NC in AH mice (Fig. 6E and F). Suppression of GSDMD or Drp1 also improved AH-induced mitochondrial injury compared with sh-NC treated AH mice by observing mitochondrial morphology (Fig. 6G). Collectively, GSDMD mediates hepatocyte pyroptosis by regulating mitochondrial function in AH.

## Discussion

Gasdermin's function in pyroptosis and inflammasome activation was initially discovered in 2015 [11, 12]. During inflammation, proteolytic cleavage of GSDMD is activated by PAMPs or DAMPs in macrophages. GSDMD cleavage releases an N-terminal fragment that can form pores on the cell membrane [17, 18]. GSDMD pores damage the integrity of the cell membrane and cause rapid pyroptotic cell death [11, 12, 19]. Growing evidence indicates that Gasdermin activation is likely to play an important role in various diseases [13, 20]. A report demonstrated that the non-canonical inflammasome pathway mediated pyroptosis, which is involved in the pathophysiology of AH [15]. They observed that the activation of Caspase 11 or Caspase 4 is increased, accompanied by increased activation of GSDMD in the livers of AH mice and patients [15]. Caspase 11 deficiency suppressed GSDMD activation, hepatocyte death, and liver injury in an AH mouse model [15]. Our results also support these findings. We found that the protein expression of GSDMD-N, NLRP3, and Caspase 11 was upregulated in the liver tissues of AH mice. The serum levels of IL-1β and IL-18 were increased in AH mice. Downregulation of GSDMD by shRNA transfection alleviated alcohol-induced hepatocyte pyroptosis and liver injury in vitro and in vivo. Therefore, targeting GSDMD activation will be a promising therapeutic approach for AH.

It has been demonstrated that mitochondrial dysfunction plays an essential role in alcohol-induced hepatocyte regeneration and liver injury [7–9]. During AH, liver cells release ROS as a direct result of alcohol intake. This leads to oxidative stress, mitochondrial damage and dysfunction, triggering inflammatory responses, cell death, and liver injury [8]. Increased production of ROS accompanied by mitochondrial DNA (mtDNA) damage was observed in mice subjected to repeated alcohol feeding [21, 22]. Increased Drp1 expression and decreased mitochondrial fusion protein (Mfn1) expression were observed in a mouse model of alcohol exposure, which is associated with mitochondrial dysfunction [23]. In this study, we found that AH could cause mitochondrial dysfunction in hepatocytes, as evidenced by the downregulation of gene expression of ND1 and ND, impaired ATP release, increased Drp1 levels and decreased Mfn1 levels in the liver tissues of mice. Studies have also shown that the expression of the Drp1 gene was upregulated in the liver tissues of patients with severe AH [24]. Further research is needed to fully elucidate the contribution of mitochondrial dysfunction to the pathology of AH in order to exploit potentially therapeutic targets.

Recent evidence has shown that GSDMD pores cause mitochondrial dysfunction during inflammation [25, 26]. In LPS-induced septic mice, GSDMD-N was upregulated in mitochondria, leading to mitochondrial dysfunction and excessive release of ROS, subsequent to the regulated activation of the NLRP3 inflammasome in the myocardium [27]. Internalized LPS can activate GSDMD and form pores, which promotes mitochondrial damage and induces mtDNA release into the cytosol [26, 28]. Our study is consistent with previous studies. In this study, we found that AH could cause mitochondrial dysfunction in hepatocytes. However, downregulation of GSDMD by sh-RNA transfection improved alcohol-induced mitochondrial dysfunction in hepatocytes in vitro. These results suggest that GSDMD activation leads to mitochondrial dysfunction in AH. We also found that improving mitochondrial dysfunction suppressed alcohol-induced hepatocyte pyroptosis by using the Drp1 inhibitor Mdivi1. Other studies have also demonstrated that Mdivi1 inhibits the NLRP3 inflammasome-mediated pyroptosis pathway and enhances mitochondrial function in various organs [29, 30]. We concluded that alcohol induces GSDMD activation, leading to mitochondrial dysfunction, which then produces ROS to activate NLRP3 and Caspase 1, subsequently causing hepatocyte pyroptosis in AH. This sequence of events forms a cascade reaction: upon activation by various stimuli, GSDMD undergoes cleavage, resulting in the formation of GSDMD-N. GSDMD-N can translocate to mitochondria, where it interacts with mitochondrial membranes, leading to the disruption of mitochondrial function. Mitochondrial dysfunction induced by GSDMD-N can lead to the overproduction of ROS within the cell. The accumulation of ROS and mitochondrial dysfunction can trigger the activation of inflammasomes, such as the NLRP3 inflammasome, leading to the activation of Caspase 1. Caspase 1 activation results in the cleavage of pro-inflammatory cytokines and GSDMD, further amplifying the inflammatory response and ultimately leading to cell pyroptosis.

Furthermore, we demonstrated that treatment with sh-GSDMD or sh-Drp1 significantly improved liver injury and mitochondrial dysfunction in AH mice. With the advancement of knowledge, pharmacological modulation of GSDMD activation and activity has the potential to become a crucial target for developing new treatments for AH. Our future research plan may focus on utilizing specific GSDMD blockers as a pharmaceutical approach for treating AH.

## Conclusions

In conclusion, this study revealed a potential mechanism by which GSDMD induces hepatocyte pyroptosis by modulating mitochondrial dysfunction during AH. These findings will not only be essential to illuminate the role of GSDMD activation-mediated mitochondrial dysfunction in AH but also pave the way to develop new therapeutic treatments for AH.

## Methods

### Reagents

F12 DMEM medium (ATCC 30-2006) and fetal bovine serum (FBS) (ATCC 30-2020) were purchased from American Type Culture Collection (ATCC, Virginia, USA). The following primary antibodies for western blot or immunohistochemistry (IHC) were as follows: ASC (DF6304, 1:1000), cleaved Caspase 1 (AF4005, 1:1000), Mitofusin-1 (Mfn1; DF7543, 1:000), pro-IL-1β (AF5103, 1:500), Mitofusin-2 (Mfn2; DF8106, 1:1000), Optic atrophy 1 (Opa1; DF8587, 1:1000), Dynamin-related protein 1 (Drp1; DF7037, 1:1000), Mitochondrial fission protein 1 (Fis1; DF12005, 1:1000), N-terminal GSDMD (DF12275, 1:1000; DF13758, 1:1000 for western blot, 1:100 for IHC), COX IV (AF5468, 1:1000) and GAPDH (AF7021, 1:10000, Affinity Biosciences) from Affinity Biosciences (Jiangsu, China); Caspase 11 (NB120-10454, 5 μg/mL for western blot, 1:200 for IHC) and NLRP3 (NBP2-12446, 2 μg/mL for western blot, 1:20 for IHC) from Novus Biologicals (Littleton, CO, USA). The secondary antibodies for western blot assay: Goat Anti-Rabbit (S0001, 1:5000 for western blot, 1:200 for IHC) and Goat Anti-Mouse (S0002, 1:5000 for western blot, 1:200 for IHC) were purchased from Affinity Biosciences; Goat Anti-Rat (ab97057, 1:10000 for western blot, 1:2000 for IHC) was obtained from Abcam (Cambridge, MA, USA). Drp1 inhibitor Mdivi1 (#34667, Sigma, USA) was dissolved in DMSO, followed diluted by culture medium. Lipofectamine 3000 reagent (L3000015) were purchased from Invitrogen (Waltham, MA, USA). GSDMD shRNA plasmid (sh-GSDMD), Drp1 shRNA plasmid (sh-Drp1) and negative control (sh-NC) were purchased from Sangon Biotechnology (Shanghai, China). Hoechst fluorescent nucleic acid stain (33342) was obtained from ImmunoChemistry technology (Bloomington, MN, USA).

### Mouse AH model

In the present study, healthy male C57BL/6 mice aged 8–12 weeks were used for all experiments. The mice were obtained from the National Laboratory Animal Center (Beijing, China). Mice were kept on a 12-h day/night cycle. All mice were given 1 week to acclimate and provided with ad libitum access to food and water. All animal experiment protocols were approved by the Institutional Animal Care and Use Committee of Peking University People’s Hospital following the guidelines (2020PHE081). AH model was induced in mice as described [15, 31]. Briefly, mice were first fed ad libitum with a high-cholesterol and high-fat diet. After 2 weeks, mice were implanted with a surgical intragastric (iG) catheter, followed by iG feeding of a high-fat diet for 1-week. Mice were then switched to an ethanol high-fat diet with ethanol concentration incrementally increasing over an 8-week period. For the knockdown of GSDMD or Drp1, AH mice were injected with sh-GSDMD or sh-Drp1 lentivirus (100 μL) via the tail vein.

### Biochemical assays

The levels of biochemical liver parameters were quantitatively measured from mouse serum using commercially available serum aspartate aminotransferase (AST), alanine aminotransferase (ALT), total cholesterol (TC), and triglyceride (TG) detection kits (Jiancheng Bioengineering Institute, Nanjing, China) following the manufacturer's instructions.

### Hematoxylin and eosin (H&E) dye

Liver fixed in a 4% paraformaldehyde solution was dehydrated and embedded in paraffin. Then paraffin sections (3 μm) were deparaffinized, hydrated, and stained with H&E dye at room temperature for 3 min. They were visualized at magnifications of 10 × and 20 × under an optical microscope (Olympus Optical, Tokyo, Japan). The histopathological scores were determined using a well-established system: (a) hepatocyte ballooning: 0 points = none, 1 point = few ballooned cells, 2 points = many cells/prominent ballooning; (b) hepatic steatosis: 0 points =  < 5% hepatocytes involved, 1 point = 5–33% hepatocytes involved, 2 points = 33–66% hepatocytes involved, 3 points =  > 66% hepatocytes involved; (c) necroinflammatory activity: 0 points = none, 1 point =  < 2 foci per 100 × field, 2 points = 2–4 foci per 100 × field, 3 points = 5–10 foci per 100 × field, 4 points =  > 10 foci per 100 × field.

### Immunohistochemistry

Firstly, cut paraffin-embedded liver tissue sections and place them on slides. Remove the paraffin and rehydrate the tissue with different grades of alcohol (100–70–50%). The slides were submerged in a boiling antigen retrieval solution for 15 min. After being washed with PBS, the slides were covered with blocking buffer for 1 h at room temperature. Then the slides were incubated with the primary antibodies (cleaved GSDMD, or NCRP3, or Caspase 11) at 4 °C overnight, followed by secondary antibodies at 37 °C for 1 h. Subsequently overlay the liver slides with diluted streptavidin visualization solution at 37 °C for 30 min. Finally, mount and coverslip the slides using a permanent mounting medium. Allow the slides to dry for 2 h before viewing them under a microscope. The slides were observed with an optical microscope (Nikon BR, Tokyo, Japan).

### Cell culture, treatment and transfection

Human hepatocyte cell line LO2 (Cell Bank, Shanghai, China) was cultured in complete RPMI-1640 medium supplemented with 10% heat-inactivated fetal bovine serum (FBS) and 1% penicillin–streptomycin. All cells were maintained in a humidified atmosphere at 37 °C supplemented with 5% CO_2_. For cell treatment, LO2 cells were plated at 6 × 10^3^ cells per well in 96-well microplates and stimulated with 40% ethanol for 48 h [32]. Where indicated, cells were pretreated the with Drp1 inhibitor Mdivi1 (10 μM) for 12 h [33]. For cell transfection, LO2 cells were transfected with sh-GSDMD or sh-Drp1 using Lipofectamine 3000 following the manufacturer’s protocol.

### Isolation of total RNA and quantitative real-time PCR (qPCR)

The RNeasy Mini Kit (Qiagen, Milan, Italy) was used for the isolation and purification of total cell or tissue RNA following the manufacturer’s instructions. The iScript cDNA Synthesis kit (Bio-Rad, Hercules, CA, USA) was used for RNA reverse transcription. After measuring the RNA concentration, 1 μg of total RNA was used to reverse transcribe to cDNA in a volume of 20 μL. To analyze the indicated gene expression, quantitative real-time PCR was performed using the Step One Plus Real-time PCR System (Applied Biosystems, Foster City, CA, USA) following the TB GreenTM Premix Ex TaqTM (TaKaRa Bio, Inc.) protocols as instructed. GAPDH as an endogenous control gene. The relative expression of target genes was calculated using the 2^−ΔΔCt^ method. The primers used in this study are listed in Table 1.

**Table 1:** Primer sequences used in this study

Gene	Primer sequence
GSDMD (human)	Forward: 5′-ATGAGGTGCCTCCACAACTTCC-3′
	Reverse: 5′-CCAGTTCCTTGGAGATGGTCTC-3′
ND1 (human)	Forward: 5′-CTCTTCGTCTGATCCGTCCT-3′
	Reverse: 5′-TGAGGTTGAGGTCTGTTAGT-3′
ND2 (human)	Forward: 5′-GTAGACAGTCCCACCCTCAC-3′
	Reverse: 5′-TTGATCCCGTTTCGTGCAAG-3′
GAPDH (human)	Forward: 5′-GTCTCCTCTGACTTCAACAGCG-3′
	Reverse: 5′-ACCACCCTGTTGCTGTAGCCAA-3′
ND1 (mouse)	Forward: 5′-GGATGAGCCTCAAACTCCAA-3′
	Reverse: 5′-GGTCAGGCTGGCAGAAGTAA-3′
ND2 (mouse)	Forward: 5′-GGCCATCGTACTCAACTATAA-3′
	Reverse: 5′-GGTAATCAGAAGTGGAATGG-3′
GAPDH (mouse)	Forward: 5′-CATCACTGCCACCCAGAAGACTG-3′
	Reverse: 5′-ATGCCAGTGAGCTTCCCGTTCAG-3′

### Western blot

At the endpoint of the experiments, lysis buffer with a protease inhibitor (P8340, Sigma) was added to the cells or liver tissues. Collected cell lysates and stored them at − 20 °C. The protein concentrations were measured using the ABC protein assay kit (Cat. No. 5000002, Bio-Rad). Protein was loaded and separated using 12% SDS-PAGE and then transferred to nitrocellulose membranes (Invitrogen). The Blots membranes were blocked with 0.01% casein at room temperature. After 1 h, the membranes were incubated with primary antibodies at 4 °C overnight. After washing with 1 × TBST, the membranes were incubated with the appropriate secondary antibodies for 1 h at room temperature. Then, blots were visualized and analyzed using the Odyssey Imaging System (LI-COR, Lincoln, NE). Representative images of three independent donors were shown in the results.

### Enzyme linked immunosorbent assay (ELISA)

Lactate dehydrogenase (LDH), IL-1β, and IL-18 levels were quantitatively measured in mouse plasma or cell supernatants using ELISA kits following the manufacturer's protocols. LDH (ml003416) kits were obtained from mlbio (Shanghai, China), IL-1β (ab100704, ab46052) ELISA kits were purchased from Abcam, and IL-18 (E-EL-M0730c, E-EL-H0253c) kits were purchased from Elabscience Biotechnology Co., Ltd (Wuhan, China).

### Hoechst 33342/propidium iodide (PI) double staining

Cell death in LO2 cells was assessed using the Hoechst 33342/PI Double Staining Kit (Roche, Basel, Switzerland) following the manufacturer's instructions. LO2 cells were fixed and labeled with a reaction mixture containing Hoechst 33342/PI dye at room temperature for 15 min. After being washed with PBS, the cells were covered with a fluorescent mounting medium. Then, PI-positive cells were counted in 5 randomly selected fields from each sample, and the percentage of PI-positive cells was calculated for statistical analysis.

### Assessment of mitochondrial membrane potential

The JC-1 staining kit (C2006, Beyotime, Shanghai, China) was used to measure the mitochondrial membrane potential of LO2 cells. Cells (2.5 × 10^5^) were resuspended in 0.5 mL of cell culture medium containing serum and phenol red. They were then incubated with 0.5 mL of JC-1 staining working solution (5×) for 20 min at 37 °C. After incubation, the cell suspension was centrifuged at 600*g* for 4 min at 4 °C to precipitate the cells. Afterward, the cell supernatant was aspirated and washed with ice-cold 1 × JC-1 staining buffer. Next, cells were resuspended by adding 1 mL of JC-1 staining buffer (1 ×), centrifuged at 600×*g* for 4 min at 4 °C, repeated twice. The precipitated cells were resuspended in 1 × JC-1 staining buffer again and analyzed with a flow cytometer (BD Biosciences, San Jose, CA, USA).

### Measurement of ROS and adenosine triphosphate (ATP) levels

The levels of ROS were measured using ROS Assay Kits (#S0033S, Beyotime, China) according to the instructions provided. Briefly, 2′,7′-dichlorodihydrofluorescein diacetate (DCFH-DA) was diluted with FBS-free cell culture medium to a 10 μM working solution. After treatment, the cells were incubated with a DCFH-DA working solution for 20 min at 37 °C. After being washed with FBS-free cell culture medium three times, the intracellular ROS fluorescence intensity was detected using a microplate reader (BioTek, Biotek Winooski, Vermont, USA). The ATP levels in cells were determined using ATP Colorimetric Assay Kits from Beyotime (#S0026) following the manufacturer's instructions.

### Transmission electron microscopy (TEM)

The liver tissues were cut into approximately 1 mm^3^ sections, and fixed in 2.5% Glutaraldehyde Fixed Solution (#G6257, Sigma) at room temperature for 1 h, followed by 4 ℃ for 1 h. After being washed with PBS, the fixed liver sections were dehydrated in graded ethanol for 15 min and then infiltrated overnight with epoxy propane embedding medium. Uranyl acetate and lead citrate were applied to post-stain ultrathin sections. The slice was observed using TEM (Hitachi, Tokyo, Japan).

### Statistical analysis

Statistical analysis of all presented data was performed using GraphPad Prism 8.0 software (GraphPad Software Inc., La Jolla, CA, USA). One-way analysis of variance (ANOVA) followed by Tukey’s multiple comparison test was applied for comparisons among multiple groups, while the unpaired *t*-test was used for comparisons between two groups. All data are presented as the mean ± standard deviation (SD) of three independent experiments conducted in triplicate. The statistical significance was set at a p-value of < 0.05.

### Supplementary Information

**Additional file 1: Figure S1.** The effects of the Drp1 inhibitor mdivi1 on hepatocytes. Hepatocytes were treated with or without 10 µM Drp1 inhibitor mdivi1 for 12 h. After stimulation, cells were collected for subsequent experiments. (A) Flow cytometry analysis of ROS using DCFH-DA in hepatocytes. (B) The levels of IL-1β and IL-18 in the culture supernatants of hepatocytes were measured by ELISA kits. (C) The expression of NLRP3, ASC, and cleaved Caspase 1 in hepatocytes was determined by western blot. Data are presented as mean ± SD. ns: no significance.

## Data Availability

The datasets used or analyzed during the current study are available from the corresponding author on reasonable request.

## Abbreviations

AH: Alcoholic hepatitis
ASC: Apoptosis-associated speck-like protein containing a CARD
ATP: Adenosine triphosphate
DAMPs: Damage-associated molecular patterns
Drp1: Dynamin-related protein 1
Fis1: Mitochondrial fission protein 1
GSDMD: Gasdermin D
Mfn1: Mitofusin-1
Mfn2: Mitofusin-2
MtDNA: Mitochondrial DNA
NLRP3: NLR family pyrin domain containing 3
Opa1: Optic atrophy 1
PI: Propidium iodide
ROS: Reactive oxygen species
TEM: Transmission electron microscopy
