# Supplementary figures and images for: GSDMD induces hepatocyte pyroptosis to trigger alcoholic hepatitis through modulating mitochondrial dysfunction
Source: Cell Div. 2024 Mar 26;19:10. doi: 10.1186/s13008-024-00114-0 (PMC10964551; doi:10.1186/s13008-024-00114-0)

**Figure S1**

**
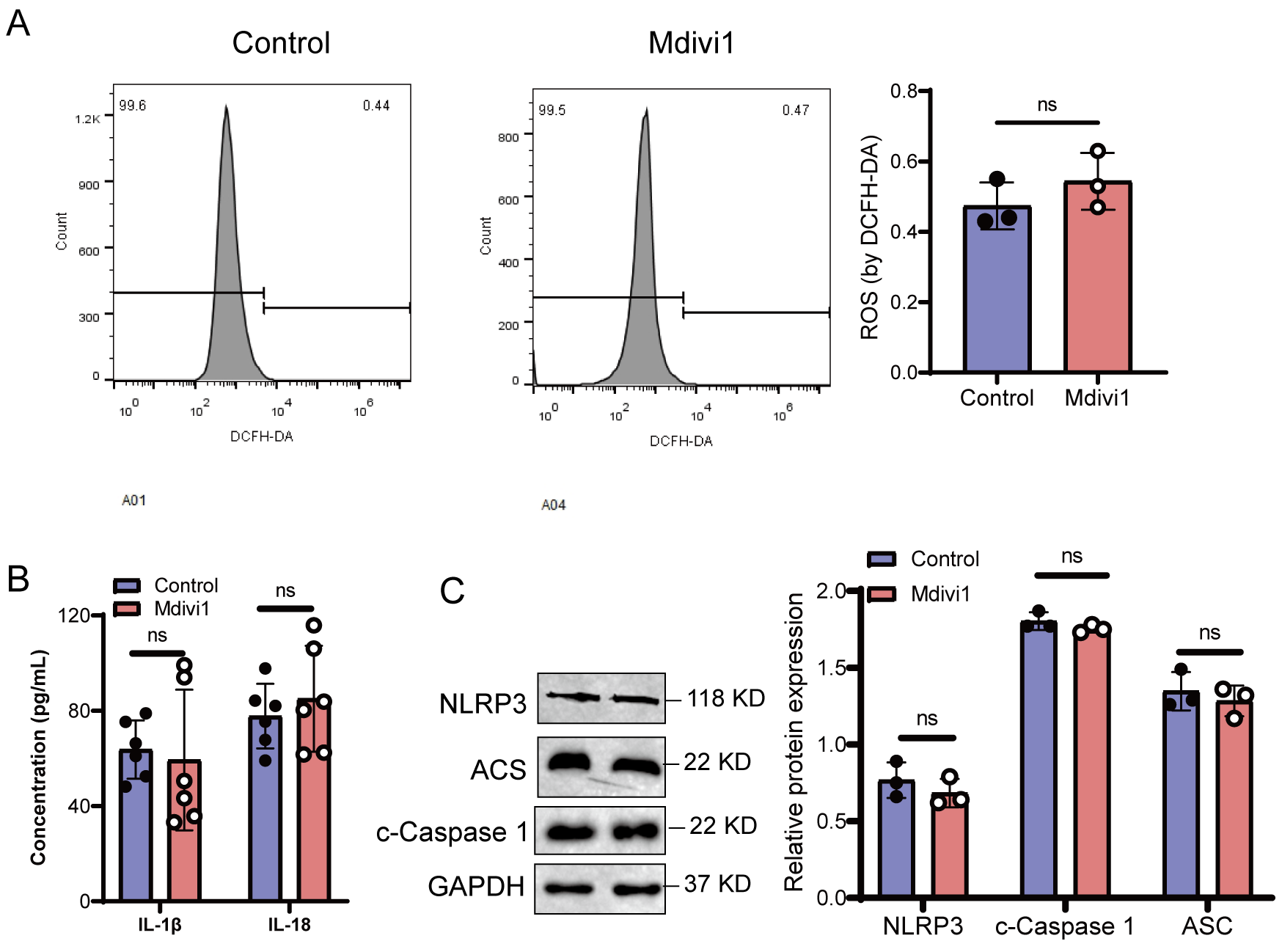
**

Supplement: Supplementary file 1 — Additional file 1: Figure S1. The effects of the Drp1 inhibitor mdivi1 on hepatocytes. Hepatocytes were treated with or without 10 µM Drp1 inhibitor mdivi1 for 12 h. After stimulation, cells were collected for subsequent experiments. (A) Flow cytometry analysis of ROS using DCFH-DA in hepatocytes. (B) The levels of IL-1β and IL-18 in the culture supernatants of hepatocytes were measured by ELISA kits. (C) The expression of NLRP3, ASC, and cleaved Caspase 1 in hepatocytes was determined by western blot. Data are presented as mean ± SD. ns: no significance. [file 13008_2024_114_MOESM1_ESM.docx]
